# Interleukin-32θ Triggers Cellular Senescence and Reduces Sensitivity to Doxorubicin-Mediated Cytotoxicity in MDA-MB-231 Cells

**DOI:** 10.3390/ijms22094974

**Published:** 2021-05-07

**Authors:** Thu-Huyen Pham, Hyo-Min Park, Jinju Kim, Jin-Tae Hong, Do-Young Yoon

**Affiliations:** 1 Department of Bioscience and Biotechnology, Konkuk University, Seoul 05029, Korea; huyenpham@konkuk.ac.kr (T.-H.P.); wkd910222@konkuk.ac.kr (H.-M.P.); jinjukim78@konkuk.ac.kr (J.K.); 2 College of Pharmacy and Medical Research Center, Chungbuk National University, Chungbuk 28160, Korea; jinthong@chungbuk.ac.kr

**Keywords:** interleukin-32θ, breast cancer, senescence, cell cycle arrest, drug resistance

## Abstract

The recently discovered interleukin (IL)- 32 isoform IL-32θ exerts anti-metastatic effects in the breast tumor microenvironment. However, the involvement of IL-32θ in breast cancer cell proliferation is not yet fully understood; therefore, the current study aimed to determine how IL-32θ affects cancer cell growth and evaluated the responses of IL-32θ-expressing cells to other cancer therapy. We compared the functions of IL-32θ in triple-negative breast cancer MDA-MB-231 cells that stably express IL-32θ, with MDA-MB-231 cells transfected with a mock vector. Slower growth was observed in cells expressing IL-32θ than in control cells, and changes were noted in nuclear morphology, mitotic division, and nucleolar size between the two groups of cells. Interleukin-32θ significantly reduced the colony-forming ability of MDA-MB-231 cells and induced permanent cell cycle arrest at the G1 phase. Long-term IL-32θ accumulation triggered permanent senescence and chromosomal instability in MDA-MB-231 cells. Genotoxic drug doxorubicin (DR) reduced the viability of MDA-MB-231 cells not expressing IL-32θ more than in cells expressing IL-32θ. Overall, these findings suggest that IL-32θ exerts antiproliferative effects in breast cancer cells and initiates senescence, which may cause DR resistance. Therefore, targeting IL-32θ in combination with DR treatment may not be suitable for treating metastatic breast cancer.

## 1. Introduction

Breast cancer is the leading cause of cancer-related deaths among women worldwide [1]. Triple-negative breast cancer (TNBC) is the most dangerous subtype of breast cancer and is associated with a significant risk of metastasis and poor overall survival [2]. Conventional genotoxic and cytotoxic chemotherapies induce death of rapidly dividing cancer cells and comprise the main strategies for treating TBNC [3]. However, due to their non-specific nature, cytotoxic strategies are also toxic to normal cells, resulting in serious side effects in various organ systems [4]. The responses of TNBC to chemotherapy are characterized by an initially high rate of complete responses and a higher relapse rate of post-chemotherapy residual tumors, unlike other types of breast cancer. Cancers are aggressive in nature and can adapt to new signaling pathways and mechanisms to escape therapeutics, especially monotherapy. Therefore, combined therapeutic modalities can be effective when responses are appropriately evaluated to eliminate drug resistance.

Cytostatic therapies are not directly cytotoxic to tumor cells, but they can reduce or prevent the growth of tumor cells by inhibiting their ability to divide [5]. Therapy-induced senescence (TIS) is a promising approach to inducing cancer cytostasis [5,6]. Senescent cells are characterized by permanent and irreversible growth arrest, changes in cell morphology, metabolism, and chromatin structure [7]. However, they also resist apoptosis and secrete various cytokines, chemokines, and growth factors (senescence-associated secretory phenotype; SASP) [8]. In addition to cytotoxic activities, some conventional therapies exhibit cytostatic activity at low doses, and promote TIS in human cancer tissues [5]. Doxorubicin (DR) is a genotoxic anthracycline drug that is commonly used to treat breast cancer; at low doses, it triggers cellular senescence in various cancer cells, including TNBC. However, drug resistance and cytotoxicity limit its effectiveness [8]. Long-term senescence may support alternative conclusions, since tumor cells can escape from TIS and facilitate drug resistance [9]. Although TIS as a strategy for treating cancer remains controversial, its primary tumor-suppressive role remains beneficial. Thus, the TIS phenomenon should be evaluated further when assessing combination therapies to help establish more effective cancer treatment strategies and prevent adverse consequences.

Interleukin (IL)- 32 has nine isoforms caused by alternative splicing [10]. Three isoforms, IL-32β, IL-32γ, and IL-32θ, have been detected in breast cancer tumors with protumor or antitumor effects [11,12]. The recently identified IL-32θ acts as an intracellular module during cancer development [12,13], and may be a potential therapeutic target. Interleukin-32θ reduces macrophage-induced breast cancer metastasis [12] and is, thus, supposed to exert antiproliferative effects on breast cancer cells. We observed that IL-32θ decreased breast cancer cell growth by inducing cellular senescence, and investigated whether this effect could help to increase cancer cell sensitivity to DR.

## 2. Results

### 2.1. IL-32θ Reduced MDA-MB 231 Cell Proliferation

**Figure 1:**
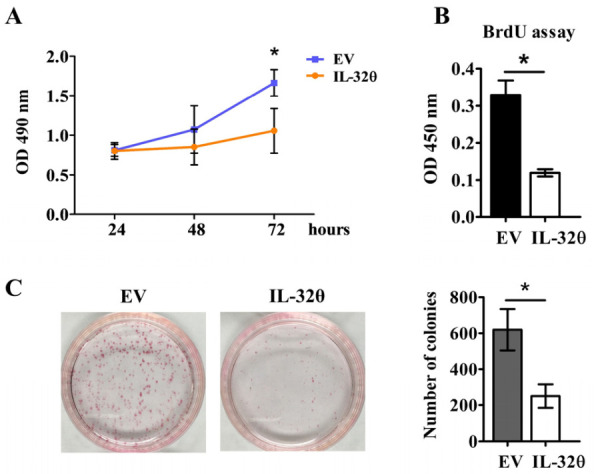
Antiproliferative effects of IL-32θ on MDA-MB-231 breast cancer cells. (**A**) Cells were seeded and incubated overnight for attachment, then cell viability was assessed using MTS assays at indicated time points. (**B**) Incorporation of BrdU after 72 h, indicating proliferative cells. (**C**) Colony forming ability was analyzed 10 days after seeding 1 × 10^3^ cells in 6-well plates. * *p* < 0.05 (Student *t*-tests). Results are representative of three independent experiments.

The results of MTS assays revealed that, after 72 h, MDA-MB 231 cells expressing IL-32θ had a slower growth rate than cells not expressing IL-32θ (Figure 1A). Moreover, DNA synthesis measured via BrdU incorporation reduced noticeably after 72 h, indicating that IL-32θ inhibited cell proliferation (Figure 1B). The results of clonogenic assays determining the colony-forming ability of single cancerous cells showed that cells expressing IL-32θ formed fewer colonies after 10 days than cells without IL-32θ expression (control) (Figure 1C). Taken together, these data suggest that IL-32θ exerted antiproliferative effects on MDA-MB-231 cells.

### 2.2. IL-32θ Induced Cellular Senescence but Not Senescence-Associated Secretory Phenotype (SASP)

**Figure 2:**
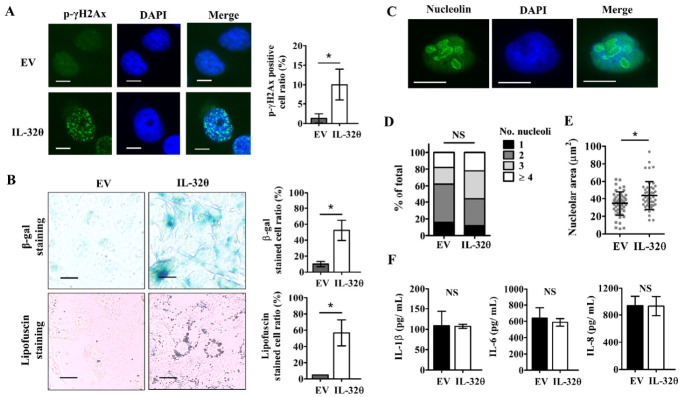
Interleukin-32θ induces senescence in breast cancer cells. (**A**) Immunofluorescence assay to detect p-γH2Ax positive cells (green). Nuclei were stained with DAPI (blue). Scale bar, 20 μm. (**B**) Ratios of cells stained with SA-β-gal or lipofuscin assessed by microscopy. Scale bar, 20 μm. (**C**) Immunofluorescence assays of nucleoli (green) with boundaries in individual nuclei determined using anti-nucleolin antibody. Scale bar, 10 μm. (**D**) Ratios (%) of total nucleoli per cell (**E**) Total area (μm^2^) of nucleoli per cell. (**F**) Secreted IL-1β, IL-6, and IL-8 measured by ELISA. * *p* < 0.05 (Student *t*-tests). Nucleoli were counted using Fiji software. Results are representative of three independent experiments. NS, not significant.

We investigated whether IL-32θ exerted antiproliferative effects on MDA-MB-231 cells by damaging DNA. The number of fluorescent cells expressing p-γH2Ax, a marker of DNA damage response, increased by ~10% compared with the total number of cells in IL-32θ-expressing cells (Figure 2A), suggesting DNA damage in some cells expressing IL-32θ. The DNA damage and reduced cell proliferation may be ascribed to cellular senescence [14]. Therefore, we detected senescence by SA-β-gal and lipofuscin staining. The ratio of stained cells was significantly higher in cells expressing IL-32θ than those without IL-32θ expression (Figure 2B). Nucleolar size is a biomarker of aging [15]. Although the proportions of nucleoli per cell did not change significantly (Figure 2C,D), the total nucleolar area per cell was larger in cells expressing IL-32θ than those without IL-32θ expression (Figure 2E). We quantified SASP that often occurs in DNA-damaged senescent cells by measuring the secretion of inflammatory IL-1β, IL-6, and IL-8 by performing the respective ELISAs (Figure 2F). The results revealed no differences between the cells with or without IL-32θ expression, indicating that SASP was not a feature of senescent cells expressing IL-32θ. Interestingly, long-term accumulation of IL-32θ in cultured breast cancer cells (>30 passages) resulted in cellular senescence, whereas short-term IL-32θ expression cells did not obviously induce senescence (Figure S1).

### 2.3. Interleukin-32θ Induced Genome Instability, Abnormal Nuclear Morphology, and Aberrant Cell Division

**Figure 3:**
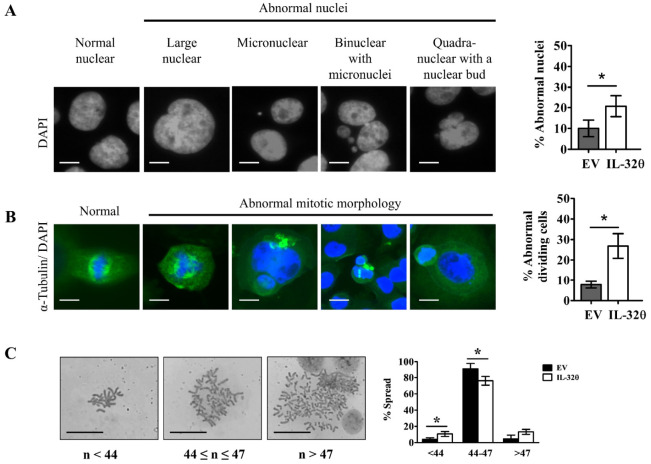
Abnormal nuclear morphology, abnormal mitosis, and genomic instability in cells with and without (control) IL-32θ expression. (**A**) Morphology of nuclei stained with DAPI assessed by fluorescence imaging. Color image was converted to grayscale to count abnormal nuclei. Scale bar, 10 μm. (**B**) Immunofluorescence assay to assess abnormal mitotic division. Cells and nuclei are stained with α-tubulin-FITC (green) and with DAPI (blue), respectively. Scale bar, 10 μm. (**C**) Chromosome spread and ratios (%) of metaphase spread (<47, 44–47, >47) in each group. Scale bar, 20 μm. * *p* < 0.05 (Student *t*-tests). Results are representative of three independent experiments.

We compared ratios of abnormal nuclei and mitotic morphology in dividing cells between the two cell lines to determine how the cells expressing IL-32θ become senescent. Abnormal nuclei are characterized by nuclear budding, blebbing, or being smaller or larger than normal size [16]. Abnormal cell division includes aberrant centromeres, missing bridges between spindle poles, and asymmetric separation [16,17]. These changes lead to unequal chromosome numbers in daughter cells, leading to aneuploidy. We noted more obvious abnormal nuclear morphology and cell division in cells expressing IL-32θ than in those without IL-32θ expression (Figure 3A,B). The results of chromosome spread assays also showed fewer chromosomes in metaphase in cells expressing IL-32θ than those without IL-32θ expression (Figure 3C). This may be ascribed to the abnormal inheritance of chromosomes from cells with micronuclei or polyploid cells during division.

### 2.4. Interleukin-32θ Induced G1 Arrest, but Not Apoptosis or Autophagy

**Figure 4:**
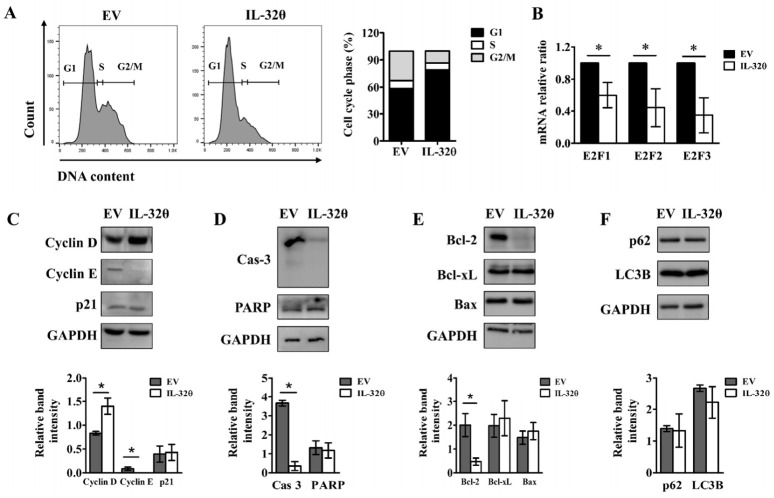
Effects of IL-32θ on cell cycle progression, apoptosis, and autophagy. (**A**) Cell cycle profiles analyzed by staining cells with PI and flow cytometry. (**B**) Messenger RNA expression of E2F1, E2F2, and E2F3, measured by qPCR. (**C**–**F**) Western blots of cell cycle-regulated proteins (Cyclin D, Cyclin E, and p21), apoptosis markers (Caspase 3 and PARP), Bcl-2 family proteins (Bcl-2, Bcl-xL, and Bax), and autophagy markers (p62 and LC3B). Proteins were quantified using Fiji software. Intensity of proteins was normalized to that of GAPDH. * *p* < 0.05 (Student *t*-tests). Results are representative of three independent experiments.

Direct consequences of genomic instability include cell cycle stress and changes in gene expression and regulation. Flow cytometry findings showed that IL-32θ induced cell cycle arrest at the G1 phase (Figure 4A). E2F transcription factors are involved in regulating G1/S transition by modulating the expression of several genes that are important for cell proliferation. Interleukin-32θ downregulated mRNA expression of the E2F factors E2F1, E2F2, and E2F3 (Figure 4B). Cyclins are core factors involved in cell cycle regulation, and we verified their expression by Western blotting. The D- and E-type cyclins mediate the G1/S phase of cell cycle progression via activating specific cyclin-dependent kinases and repressing E2F-mediated transactivation of S-phase genes [18]. Cyclin D1 is overexpressed in various types of cancer and is associated with tumorigenesis and metastasis [18]. Loss of cyclin D causes G1 arrest, but cyclin E can replace cyclin D to facilitate G1/S progression in a different cellular context. G1/S transition is regulated by a restriction point (R point), and the postmitotic G1 cells that do not reach the R point do not exhibit cyclin E accumulation [19]. We found that the expression of cyclin D1 was significantly increased, whereas that of cyclin E1 was reduced in cells expressing IL-32θ (Figure 4C). Thus, IL-32θ-expressing cells were probably permanently arrested in the late G1 phase, irrespective of p21 expression status, because p21 protein expression did not differ between the two cell lines (Figure 4C). To identify whether IL-32θ could induce other types of cell death, along with senescence, we examined protein expression of the apoptosis markers caspase 3, poly (ADP-ribose) polymerase (PARP), and Bcl-2 family proteins that regulate cell death via modulating mitochondrial outer membrane permeabilization (MOMP), as well as the expression of autophagy markers (p62 and LC3). As shown in Figure 4D,E, expressions of Bcl-2 and caspase 3 protein were downregulated, suggesting apoptosis cell death through mitochondrial outer membrane permeabilization (MOMP). However, the activation of PARP as a downstream factor of caspase 3 and the protein expression levels of pro-apoptotic Bax and anti-apoptotic Bcl-xL were not altered in IL-32θ-expressing cells (Figure 4D,E). Moreover, the cleavage of LC3 and upregulated expression of p62 were not detected (Figure 4F). Therefore, the results revealed that the apoptotic and autophagic pathways were not related with IL-32θ-inhibited cell proliferation, although mild MOMP was observed in IL-32θ-expressing cells.

### 2.5. IL-32θ Maintained Senescence and Resisted against DR-Induced Cell Death

**Figure 5:**
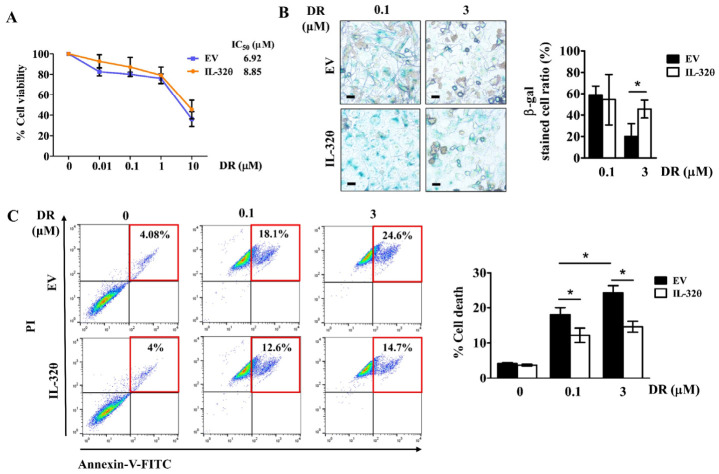
Effects of IL-32θ on doxorubicin-treated breast cancer cells. (**A**) Cell viability quantified by MTS assays and IC_50_ of different doses of DR. (**B**) Cells positive for SA-β-gal and count ratios (%). Scale bar, 20 μm. (**C**) Ratios (%) of cell death measured by Annexin-V/PI staining, followed by flow cytometry. * *p* < 0.05 (Student *t*-tests). Results are representative of three independent experiments.

Doxorubicin treatment significantly reduces the viability of breast cancer cells after 48 h [20,21,22]. We assessed the effects of DR on the viability of MDA-MB-231 cells with and without IL-32θ expression for 24 h and determined whether IL-32θ contributes to DR toxicity in these cells. The results showed that the viability of MDA-MB-231 cells decreased depending on the DR dose. The IC_50_ values of the control and IL-32θ-expressing cells were 6.92 and 8.85 μM, respectively (Figure 5A), suggesting that IL-32θ reduces DR toxicity on breast cancer cells. Doxorubicin induces senescence at a low dose and induces cell death at high doses, respectively [5]; thus, we compared both cell lines under treatment at low (0.1 μM) and high (3 μM) doses of DR. We found senescent cells clearly among the control cells incubated with a low but not a high dose of DR, and in cells expressing IL-32θ incubated with both DR doses (Figure 5B). The numbers of dead cells verified by annexin-V FITC/PI staining gradually and dose-dependently increased in the controls, but did not alter significantly between 0.1 and 3 μM DR treatment in cells expressing IL-32θ (Figure 5C). Moreover, the ratio of cell death was higher, regardless of the DR dose in control, than in IL-32θ-expressing cells. These results suggested that senescent cells expressing IL-32θ were stable and resisted DR-induced cell death.

### 2.6. Interleukin-32θ Supports Ploidy Formation and G2/M Arrest in Cells Incubated with 0.1 μM DR

**Figure 6:**
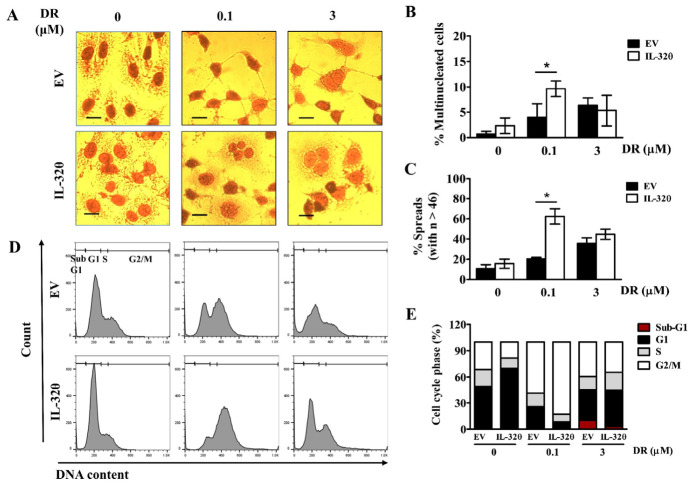
Effects of IL-32θ on morphology, genome stability, and cell cycle progression in cells incubated with doxorubicin (DR). (**A**) Morphology of cells stained with hematoxylin and eosin. Scale bar, 10 μm. (**B**) Cells with more than one nucleus are counted as multinucleated. (**C**) Ratios (%) of metaphase spreads with >46 chromosomes. Scale bar, 20 μm. (**D**,**E**) Cell cycle phases determined by PI staining and flow cytometry. * *p* < 0.05 (Student *t*-tests). Results are representative of three independent experiments.

Low doses of DNA damaging drugs such as DR can induce mitotic catastrophe, genomic instability, and senescence, during which cells become giant, polyploid, and multinucleated [23,24]. Polyploidy is a common feature of aggressive cancer cells with genomic instability, and it is characterized by the presence of more than two basic sets of chromosomes in cells [25]. Incubation with 0.1 μM DR resulted in a sharp increase in the number of multinucleated cells expressing IL-32θ (Figure 6A,B), and a higher ratio of cells with large metaphase spreads with >46 chromosomes compared with the control (Figure 6C). Doxorubicin arrests MDA-MB-231 cells only at the G2/M checkpoint [26]. We also found that the ratio of G2/M arrest was significantly higher in cells expressing IL-32θ than in control cells (Figure 6D,E). A high dose (3 μM) of DR released G2/M arrest and cells expressing IL-32θ appeared to re-enter the cell cycle, whereas control cells started to undergo apoptosis due to the high ratio of the cells at the sub-G1 phase (Figure 6D,E). Collectively, our data suggested that IL-32θ expression promoted endoreplication in MDA-MB-231 cells because of the high ratio of G2/M arrest and ploidy formation at a low dose of DR, and endomitosis by re-entering the cell cycle at a high dose of DR.

## 3. Discussion

Cellular senescence is related to irreversible growth arrest and can arise due to activated oncogenes, or be prematurely induced by stress [27]. Common features of these senescence types are cell cycle arrest at the G1 phase, DNA damage, enhanced SA-β-gal activity, increased size, and granulated cells [8]. Senescent cells become aging, grow slowly, and can be cleared by immune cells [28]. Cancer cells attempt to survive by avoiding senescence. Senescent cancer cells become giant, polyploid, and poorly recognized [24]. Ploidy formation is the result of compromised aberrant checkpoints or abnormal mitosis and endoreplication. Giant senescent cells cannot propagate and mostly stop proliferating [24]. However, polyploid giant cancer cells (PGCCs) exit mitosis and divide into aneuploid cancer cells that can grow and form a new generation of cells with abnormal characteristics [25]. Therefore, cells undergoing senescence, which can occur at an early stage of cancer, can still support tumorigenesis. We found that IL-32θ induced senescence via inducing permanent cell cycle arrest at the G1 phase, thus significantly reducing the growth rate of MDA-MB-231 cells. Senescent cells expressing IL-32θ also showed increased genomic instability, nuclear instability, and aberrant mitosis, although polyploid cells were not distinguishable. These abnormalities accumulating over a long term could be the main cause of aneuploid cell formation. Cells expressing IL-32θ may have dual and opposing actions, including antiproliferative effects and the promotion of aneuploid cells.

Senescent cells can adversely affect the tissue microenvironment [29,30,31]. The most important of these effects is the acquisition of a senescence-associated secretory phenotype (SASP), which converts senescent cells into proinflammatory cells that secrete various growth factors, cytokines, and chemokines to promote tumor progression [31]. Inhibiting the production of specific SASP factors suppresses tumorigenesis and the immunosuppressive environment promoted by senescent cancer cells [31]. Moreover, SASP inhibition may reduce the risk of some senescent cancer cells escaping growth arrest and resuming abnormal progression. Our data revealed no significant changes in the SASP secretory factors IL-1β, IL-6, and IL-8 in cells expressing IL-32θ compared with controls. Studies targeting macrophage-secreted chemokines have shown that IL-32θ exerts anti-inflammatory effects on macrophages and anti-metastatic effects on breast cancer [12,32,33]. Although senescent cells without SASP characteristics may show less pro-tumorigenic phenotype, these tenacious cells can escape immune clearance and turn resistant to anti-cancer agents. Senescent growth arrest without SASP depends on either the p21- or the p16INK4a-mediated pathway [31]. We found no relationship between IL-32θ and p21 expression, and p16INK4a expression could not be verified due to a deletion of the INK4/ARF locus in the MDA-MB-231 chromosome [34].

Doxorubicin is not always effective, and drug resistance can occur due to polyploidization [23,24]. Low doses of DR lead to mitotic catastrophe, followed by endoreduplication and polyploidy formation [23,24]. Tumor cells escape DR-induced senescence and can become more malignant [5]. Doxorubicin induces senescence and the expression of the SASP factors that mediate cancer relapse by stimulating the malignant transformation of surrounding non-senescent breast cancer cells in MMTV-Wnt1 mouse models of breast carcinoma [35]. Genetic and pharmacological clearance of DR-induced senescent cells from an orthotopic model of breast carcinoma reduces cancer growth and limits cancer relapse [35]. We found that a low dose of DR caused cells expressing IL-32θ to exhibit multinucleation and become polyploid compared with controls. This could explain why cells expressing IL-32θ resisted DR and had delayed cell death.

To design new agents targeting specific pathways associated with tumor growth or metastasis, preclinical and clinical information is needed to understand the factors determining drug sensitivity or resistance. Demand is increasing for experimental data that could guide the administration of current cytotoxic agents combined with novel targeting drugs. For example, DR can synergize with the Src inhibitor (dasatinib) or the Hsp90 inhibitor (gamitrinib) to block breast cancer cell growth [36,37]. The effects of DR combined with other therapeutic drugs, at the level of cell cycle regulation, apoptosis, and transcriptome, are not completely understood and should be further investigated to gain insights into the mechanisms of combinatorial strategies for treating breast cancer. We previously revealed the anti-metastatic effects of IL-32θ on breast cancer cells under macrophage stimulation [12]. Here, we aimed to determine whether IL-32θ could synergize with genotoxic DR. Our findings showed that IL-32θ could not synergize with genotoxic DR, indicating that this combination cannot prevent breast cancer. The limitation of our study is the use of single metastatic breast cancer cell line. Moreover, we could not generate any model cells overexpressing IL-32θ using p53 wild-type cancer cells because the cells could not proliferate or expressed no detectable IL-32θ protein (data not shown). We speculate that wild-type p53 may negatively contribute to the IL-32θ regulated mechanism, which should be further investigated. Further in vivo models would be ideally designed and assessed to support the in vitro results in the study of combination therapies.

## 4. Materials and Methods

### 4.1. Cell Culture

Breast cancer MDA-MB-231 (ATCC^®^ HTB-26™, Manassas, VA, USA) cells with and without stable expression of IL-32θ [12] were cultured in DMEM (Hyclone Laboratories, Logan, UT, USA) supplemented with 10% heat-inactivated fetal bovine serum (Gibco, NY, USA), 100 units/mL of penicillin, and 100 μg/mL of streptomycin, and maintained at 37 °C under a 5% CO_2_ atmosphere until they reached confluence. Cultures were regularly tested for mycoplasma contamination. Doxorubicin hydrochloride (PubChem Substance ID 57650234) was purchased from MilliporeSigma, Burlington, MA, USA.

### 4.2. Clonogenic Assay

Cells (1 × 10^3^) were seeded in 6-well plates and incubated for 10 days to form viable colonies, which were then fixed in 4% paraformaldehyde for 15 min, washed with phosphate buffered saline (PBS), and stained with hematoxylin for 3 min and eosin for 1 min. The plates were washed with distilled water and dried at room temperature (RT). Colonies were counted using Fiji, an ImageJ open source software [38].

### 4.3. Cell Viability Assay

Cell viability was assessed using CellTiter 96 ^®^AQueous One Solution Cell Proliferation Assays (Promega Corp., Madison, WI, USA). The MDA-MB-231 cells (10^4^/100 μL) were seeded in 96-well plates and incubated for 20 min with 20 μL of 3-(4,5-dimethylthiazol-2-yl)-5-(3-carboxymethoxyphenyl)-2-(4-sulfophenyl)-2H-tetrazolium (MTS), with the electron coupling reagent phenazine methosulfate (PMS). Optical density (OD) was measured at 490 nm. Relative viability was quantified by normalization to the control OD.

### 4.4. Reverse Transcription Quantitative PCR (RT-qPCR)

We measured mRNA expression in MDA-MB-231 cells using RT-qPCR. Total RNA from cells was extracted using the Easy-BLUE reagent (iNtRON Biotechnology, SungNam, Korea), then RT-PCR was performed using SensiFAST™ SYBR NO-ROX Kit (Bioline, London, UK) and results were analyzed using Rotor-Gene 6000 series software 1.7 (Qiagen, Hilden, Germany). The sequences of primer sets for E2F1, E2F2, E2F3 were referred from [39]. mRNA expression levels were calculated using the ΔΔCt method [40].

### 4.5. Western Blotting

Cells were harvested and lysed using radioimmunoprecipitation assay (RIPA) buffer (DyneBio, Seoul, Korea) containing 1 × protease and phosphatase inhibitor cocktail (Roche Diagnostics, Mannheim, Germany). Samples were separated by 10% SDS-PAGE, transferred to polyvinylidene difluoride (PVDF) membranes (GE Healthcare, Buckinghamshire, UK), and incubated with primary antibodies at 4 °C overnight. After that, membranes were washed by Tris-buffered saline containing 0.05% Tween-20 (TBST), then incubated with secondary antibodies for 1 h. Blots were visualized using chemiluminescence detection kits (Advanstar, Cleveland, OH, USA) and detected using an EZ-capture MG protein imaging system (ATTO, Tokyo, Japan). Proteins in the blots were quantified using Fiji software and normalized to GAPDH. Primary antibodies include antibodies against p21, caspase-3, PARP, Bcl-2, Bcl-XL, Bax (Cell Signaling Technology, Danvers, MA, USA); cyclin D, cyclin E, GAPDH (Santa Cruz Biotechnology, Dallas, TX, USA); p62, LC3B (MilliporeSigma). Secondary antibodies include anti-rabbit IgG antibody (Bethyl Laboratories Inc., Montgomery, TX, USA) or anti-mouse IgG antibody (Santa Cruz Biotechnology) conjugated to horseradish peroxidase (HRP).

### 4.6. Cell Cycle Analysis and Apoptosis Detection by Flow Cytometry

For cell cycle analysis, MDA-MB-231 cells were washed with PBS, and suspended in hypotonic buffer containing propidium iodide, as described in [41]. For detection of apoptosis, cells were stained using FITC-Annexin V Apoptosis Detection Kits (BD Biosciences, San Jose, CA, USA), as per manufacturer’s protocol. Fluorescence emission was measured using a FACSCalibur flow cytometer (BD Biosciences) and analyzed using FlowJo software (Tree Star Inc., Ashland, OR, USA).

### 4.7. Cell Proliferation Assay (BrdU)

Cell proliferation was determined based on 5′-bromo-2′-deoxyuridine (BrdU) incorporation into DNA using Cell Proliferation Assay Kits (Cell Signaling Technology, Danvers, MA, USA), following the manufacturer’s protocol. Cells (10^4^ cells/mL) were seeded in 96-well plates, cultured for 3 days, and labeled with BrdU. The cells were fixed, then bound antibody was detected using an HRP-linked antibody. Color was developed using the HRP substrate, 3,3′,5,5′-tetramethylbenzidine (TMB), and the OD was assessed at 450 nm. The OD reflects the amount of BrdU incorporated into the cells, which is a direct indication of cell proliferation.

### 4.8. Enzyme-Linked Immunosorbent Assay (ELISA)

Amounts of cytokines and chemokines were analyzed in cell culture supernatants using human IL-1β, IL-6, IL-8 ELISA kits (R&D Systems, Minneapolis, MN, USA), according to the manufacturer’s protocol.

### 4.9. Immunofluorescence Assay

Cells (10^5^ cells/mL) were seeded on glass chamber slides (SPL, Pocheon, Korea) overnight. Attached cells were fixed in 4% paraformaldehyde, permeabilized with cold methanol, and nonspecific protein binding was blocked for 1 h at RT with 0.1% bovine serum albumin in PBS. Primary antibodies (1:200) were added to the chamber slides and incubated at 4 °C overnight. After washing with PBS, the chamber slides were incubated with goat anti-mouse IgG (H + L) FITC conjugated (1:400 dilution) (Millipore, Temecula, CA, USA). Nuclei were stained with 1 μg/mL of 4, 6-diamidino-2-phenylindole (DAPI) (MilliporeSigma) for 30s, then the cells were visualized using the EVOS M7000 Imaging System (Thermo Fisher Scientific Inc., Waltham, MA, USA). The area of each nucleolus was quantified by measuring a cross-sectional area corresponding to nucleolin stain using Fiji software, as described in [15]. Primary antibodies include nucleolin, phosphor S319-γH2Ax (Cell Signaling Technology), and α-tubulin (Santa Cruz Biotechnology).

### 4.10. Senescence-Associated β-Galactosidase (SA-β-gal) Assay

We seeded MDA-MB-231 cells (10^5^ cells/mL) in chamber slides to attach overnight. The cells were stained using a senescence β-galactosidase staining kit (Cell Signaling Technology). Senescent cells were visualized under an inverted microscope (Zeiss Primo Vert, Göttingen, Germany) and counted in three random areas. Senescent cells were also detected based on lipofuscin staining using Alfa Aesar™ Sudan Black B (Thermo Fisher Scientific Inc.), as described earlier [42].

### 4.11. Chromosome Spread Assay

Cells were incubated at 37 °C for 45 min with KaryoMAX™ Colcemid™ (Thermo Fisher Scientific Inc.) at a final concentration of 0.1 μg/ mL. The cells were trypsinized, suspended in hypotonic 0.075 M KCl at 37 °C for 10 min, and fixed in methanol–acetic acid buffer (3:1). The fixed cells were dropped onto slides, dried for 1 h, and stained with Giemsa (MilliporeSigma). Metaphase spread was captured under a Zeiss Primo Vert optical microscope. At least 20 metaphases per sample were used for statistical analysis.

### 4.12. Statistical Analysis

Data are presented as means ± standard deviation (SD). Two groups were compared using Student *t*-test. Data were statistically analyzed using Prism version 5.0. (GraphPad Software Inc., San Diego, CA, USA) All *p*-values were two-sided, and results with *p* < 0.05 were considered statistically significant.

## 5. Conclusions

We showed that IL-32θ exerted antiproliferative effects on breast cancer cells by inducing senescence and permanent cell cycle arrest. The genomes of cells expressing IL-32θ became unstable, with a higher ratio of abnormalities than control cells. The prevalence of polyploidy increased significantly among cells expressing IL-32θ that were incubated with low-dose DR, while cell death was delayed at a high dose of DR. Therefore, IL-32θ overexpression combined with DR may not be beneficial for treating patients with breast cancer.

## Data Availability

Data are contained within the article and supplementary material.

## Supplementary Materials

The following are available online at https://www.mdpi.com/article/10.3390/ijms22094974/s1. Figure S1: IL-32θ induce cellular senescence at late stage (>30 passages) but not early stage (<30 passages).

## Abbreviations

DR	Doxorubicin
EV	Empty vector
MOMP	Mitochondrial outer membrane permeabilization
PGCCs	Polyploid giant cancer cells
SASP	Senescence-associated secretory phenotype
TIS	Therapy-induced senescence
TNBC	Triple-negative breast cancer
