# Supplementary material for: Interleukin-32θ Triggers Cellular Senescence and Reduces Sensitivity to Doxorubicin-Mediated Cytotoxicity in MDA-MB-231 Cells
Source: Int J Mol Sci. 2021 May 7;22(9):4974. doi: 10.3390/ijms22094974 (PMC8124300; doi:10.3390/ijms22094974)
Supplement: Supplementary file 1 [file ijms-22-04974-s001.zip › ijms-1204386-supplementary.pdf]

Supplementary data

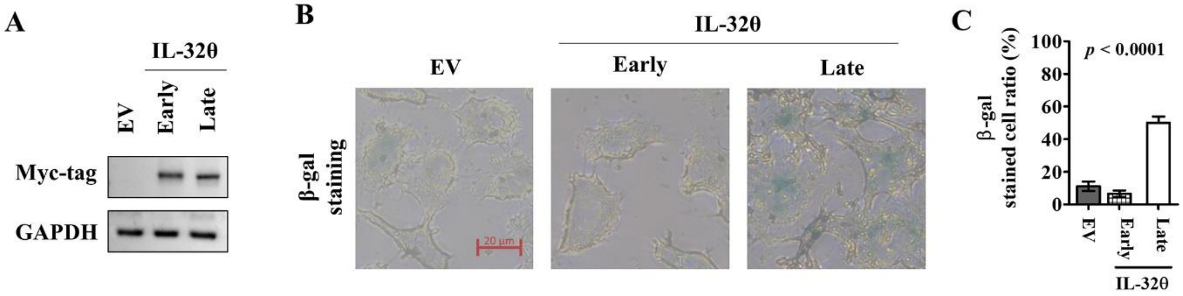

Figure S1. IL-32θ induce cellular senescence at late stage (> 30 passages) but not early stage (< 30 passages). (A) Constitutive expression of IL-32θ in MDA-MB-231 cells at early and high passages verified by Western blot analysis using Myc-tag antibody. (B) Cellular senescent control cells, IL-32θ expressing cells at early and late stage stained with SA-β-Gal assessed by microscopy. Scale bar, 20 μm. C) Ratios of cells stained with SA-β-Gal. $P < 0.0001$ (One way ANOVA test followed by Tukey's HSD test). Results are representative of three independent experiments.
